# Roux-en-Y Gastric Bypass as Conversion Procedure of Failed Gastric Banding: Short-Term Outcomes of 1295 Patients in One Single Center

**DOI:** 10.1007/s11695-023-06746-5

**Published:** 2023-08-07

**Authors:** Karen Handojo, Aiman Ismaeil, Andries Van Huele, Christophe Van Neste, Isabelle Debergh, Bruno Dillemans

**Affiliations:** 1 Department of General Surgery, AZ Sint Jan Brugge-Oostende AV, Campus Henri Serruys, 8400 Oostende, Belgium; 2 Department of General Surgery, AZ Sint-Jan Brugge-Oostende AV, Ruddershove 10, 8000, Brugge, Belgium; 3 Department of General Surgery, Faculty of Medicine, Aswan University, Aswan, 81528 Egypt; 4 Department of Urology, Ghent University Hospital, Ghent, Belgium; 5 Meise Botanical Garden, Nieuwelaan 38, 1860, Meise, Belgium; 6 Department of General Surgery, AZ Delta Hospital, Sint-Rembertlaan 21, 8820, Torhout, Belgium

**Keywords:** Revisional bariatric surgery, Conversion, Gastric banding, Gastric bypass, One-step, Two-step

## Abstract

**Purpose:**

Laparoscopic adjustable gastric band (LAGB) has high technical and weight loss failure rates. We evaluate here the 1-year morbidity, mortality, and weight loss of laparoscopic Roux-en-Y-gastric bypass (LRYGB) as a feasible conversion strategy.

**Methods:**

Patients with a failed primary LAGB who underwent LRYGB from July 2004 to December 2019 were selected from an electronic database at our center. Patients had a conversion to LRYGB at the same time (one-stage approach) or with a minimum of 3 months in between (two-stage approach). Primary outcomes included 30-day morbidity and mortality. Secondary outcomes were body mass index (BMI), percent excess weight loss (%EWL), and percent excess BMI lost (%EBMIL) at 1 year postoperatively.

**Results:**

A total of 1295 patients underwent a conversion from LAGB to LRYGB at our center: 1167 patients (90.1%) in one stage and 128 patients (9.9%) in two stages. There was no mortality. An early (30-day) postoperative complication occurred in 93 patients (7.2%), with no significant difference found between groups. Hemorrhage was the most common complication in 39 patients (3.0%), and the reoperation was required in 19 patients (1.4%). At 1 year postoperatively, the mean BMI was 28.0 kg/m^2^, the mean %EWL 72.8%, and the mean %EBMIL 87.0%. No statistically significant difference was found between the groups.

**Conclusion:**

Conversion to LRYGB can be considered as a safe and effective option with low complication rate and good weight loss outcomes at 1 year. One-stage conversion provides the same early outcome as two-step surgery with a competent surgeon.

**Graphical Abstract:**

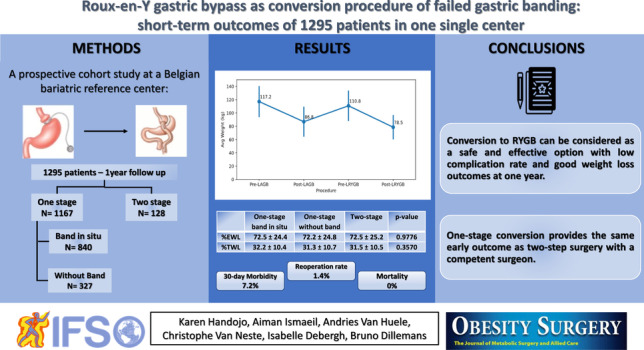

## Introduction

Laparoscopic adjustable gastric banding (LAGB) was very popular in the late 1990s, being the most frequently performed procedure worldwide. Nowadays, it has diminished in popularity despite being a minimally invasive and reversible procedure with low short-term morbidity [1]. According to the IFSO Worldwide Survey, LAGB was performed in 24.4% of all bariatric procedures in 2003, with a peak (42.3%) in 2008 and a substantial decrease (7.4%) in 2014 [2, 3]. This is due to insufficient long-term weight loss and a high rate of technical failure associated with LAGB [4–7], which implies revisional surgery for a substantial number of patients. Currently, laparoscopic Roux-en-Y-gastric bypass (LRYGB) and laparoscopic sleeve gastrectomy (LSG) have by far surpassed the LAGB in the ranking of primary bariatric surgical procedures across the globe [8].

Although inadequate results from LAGB are best managed with removal and conversion to an alternative bariatric procedure [9], there is no consensus regarding the best surgical options for revision. Several interventions have been described, with LRYGB or LSG as the most frequent [10].

The aim of this large single-center study is to evaluate the short-term morbidity, mortality, and weight loss at 1 year in patients who underwent LRYGB as a conversion procedure of unsuccessful LAGB.

## Patients and Methods

In our center, all patients who underwent LRYGB after failed LAGB were prospectively enrolled in an electronic database as from July 2004. Patient data were gathered through intensive research in the medical record database along with telephone interviews. The study was approved by the Ethical Commission of the AZ Sint Jan Hospital in Bruges, Belgium (B2020049000027, 25/11/2020) and conducted in accordance with the principles of the Declaration of Helsinki. The first results were published previously in 2016 to compare one-step versus two-step approach of conversion [11]. All patients gave written informed consent. For the current study, the patients were divided into 2 groups: those with LAGB in situ and conversion to LRYGB at the same time (one-stage approach), or with a minimum of 3 months in between (two-stage approach). At the end, we added a third group, in which the band was removed prior to revision, since the LRYGB in those instances could be as challenging as when the band was still in place (i.e., fibrotic tissue, non-division of the gastro-gastric sutures, multiple adhesions). Follow-up was planned after 6 weeks, 6 months, and 1 year. Lost to follow-up were patients who had insufficient follow-up data at the 1-year consultation or could not be reached by telephone.

### Surgical Technique

All procedures in this study were performed under the supervision of one single surgeon (B. Dillemans), following a standardized and previously published surgical approach [11, 12]. In summary, the band was removed if it was still in place, and the fibrous capsule and gastro-gastric sutures were opened. One-stage conversion was the first option, unless this was considered unsafe based on pre- or perioperative findings, i.e., band migration or iatrogenic gastric lesions during the dissection. The horizontal transection of the pouch started distal to the previous band position, except in cases of a grossly dilated pouch. The gastric pouch was completed vertically with linear staplers. The gastrojejunal anastomosis was constructed using a 25-mm circular stapler, in an ante-colic, ante-gastric way. A side-to-side-linear stapled jejunojejunostomy was constructed with an alimentary limb measuring 130 cm and a biliopancreatic limb of 50 cm. In patients with a BMI < 35 kg/m^2^, an alimentary limb of 75 cm was made with a normal biliopancreatic limb. In case of BMI > 45 kg/m^2^, the alimentary limb measured 75 cm, while the biliopancreatic limb was increased to 150 cm. In very selected cases (BMI > 45 kg/m^2^, no reflux esophagitis, age < 40 years old, and with informed consent), the band was kept in place and repositioned around the newly constructed gastric pouch.

### Outcome Measures

**Fig. 1:**
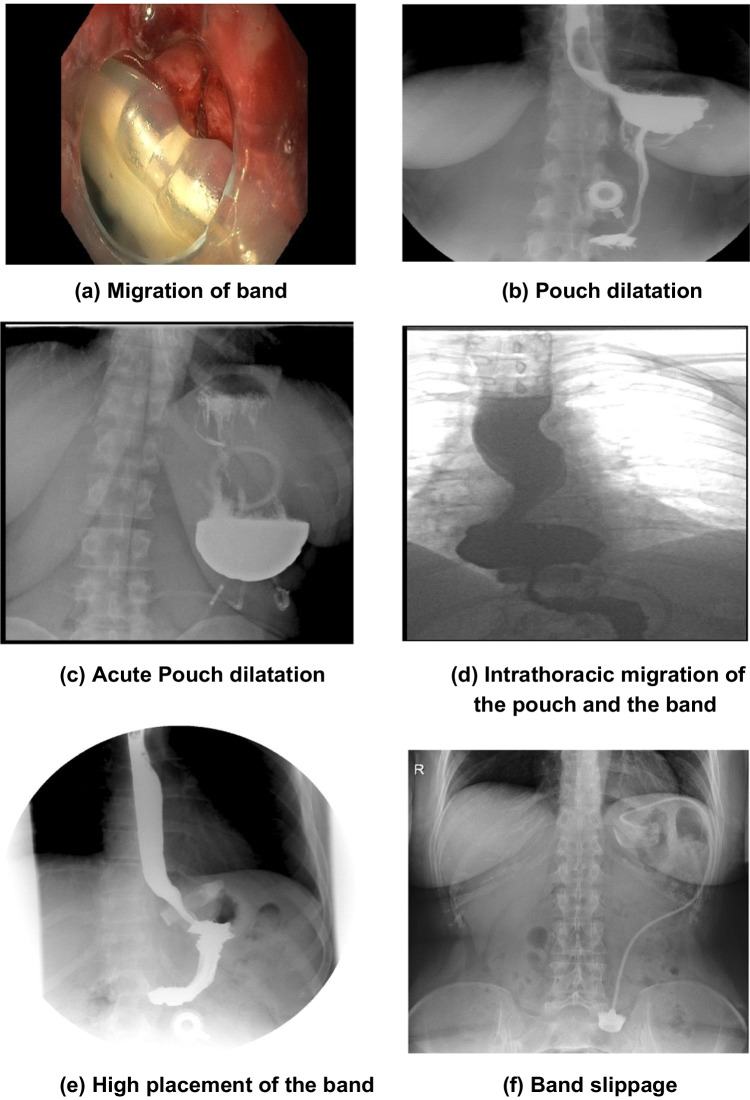
Band-related complications

The following data were assessed:

1. Patient demographics and medical data: including age, gender, height, country of origin, technical details of operation, hospital stay, weight before LAGB, date of LAGB, date of LAGB removal, date of revisional procedure, weight before conversion to RYGB, and weight at last follow-up.
2. The reason for conversion: i.e., unsatisfactory weight loss or weight regain, or band-related complications (i.e., band slippage or erosion, recurrent port tilting, port infection), or both (Fig. 1).
3. Primary outcomes include 30-day morbidity and mortality. Major complications were defined as any complication that resulted in prolonged hospital stay (>7 days), venous thrombotic event requiring administration of a therapeutic anticoagulant, reoperation, or re-intervention. Minor complications include everything that is not included under major, according to the recommendations of the ASMBS [13].
4. Initial body mass index (BMI), change in BMI (∆BMI), percent excess weight loss (%EWL), percent total weight loss (%TWL), and percent excess BMI loss (%EBMIL) were calculated using the following measures:
  Initial mean BMI = Initial weight/height^2^
  ∆BMI = (Initial BMI) − (Postop BMI)
  %EWL = {(Initial weight) − (Postop weight)}/{(Initial weight) − (Ideal weight)} × 100
  % EBMIL = {Change in BMI/(Initial BMI − 25)} × 100
  % TWL = {(Initial weight) − (Postop weight)}/{(Initial weight)} × 100

Weight was calculated in kilograms and height in meters.

### Statistical Analysis

Statistical analysis was done with Python Packages SciPy and Stats models. Quantitative variables were compared with SciPy Anova function and qualitative variable comparison performed with Stats models chi-squared test. For both types of analysis, a *p* value below 0.05 was considered significant.

## Results

### Preoperative Demographics

**Fig. 2:**
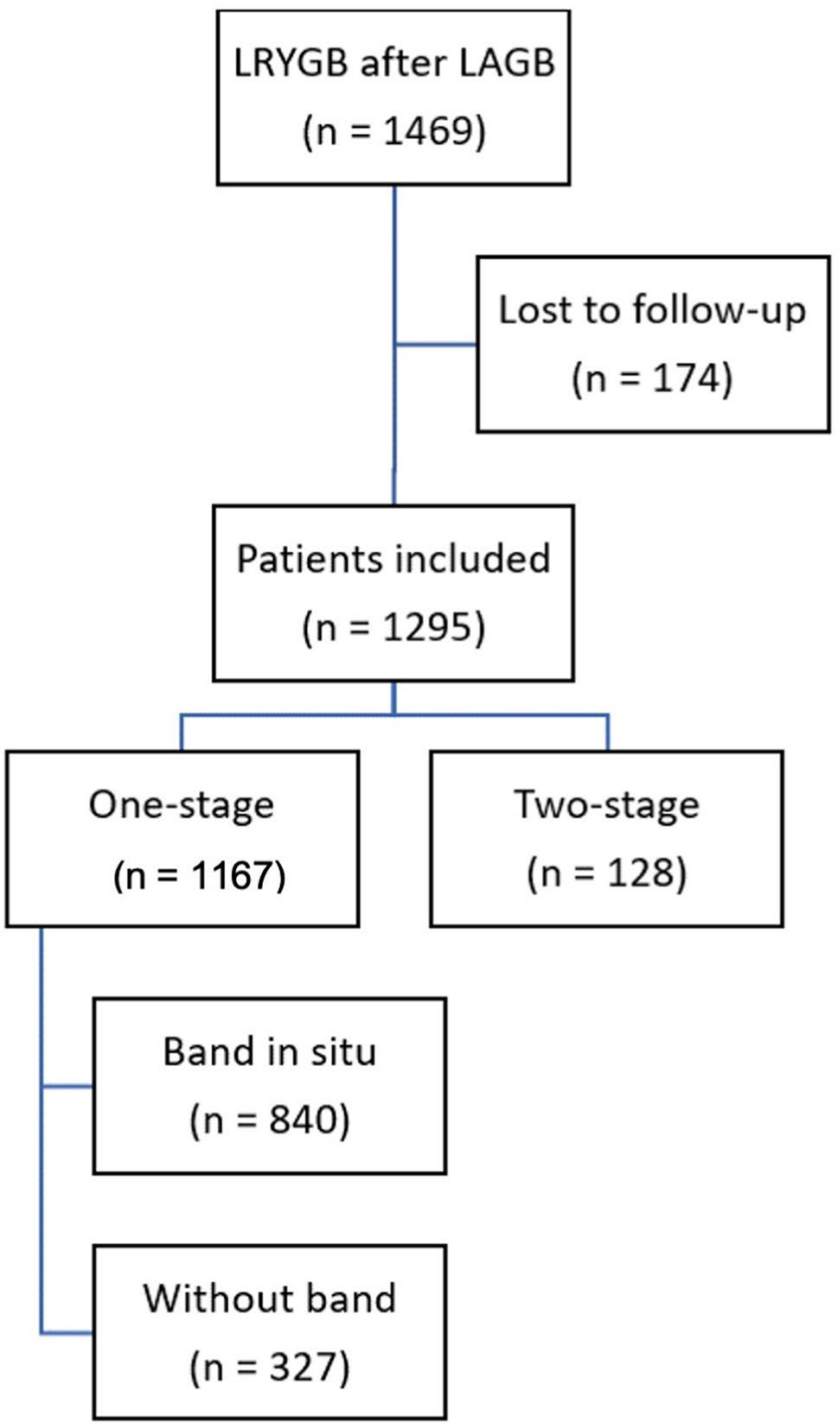
Patient flow chart. *LRYGB* laparoscopic Roux-en-Y gastric bypass, *LAGB* laparoscopic gastric banding

Between July 2004 and December 2019, a total of 1469 patients underwent a conversion from LAGB to RYGB (Fig. 2). Of these, we were able to obtain 1-year follow-up in 1295 patients (88%). The majority of patients (82.8%) were female. The mean age of the patients at the time of conversion was 44.2 ± 10.3 years. More than half of our cohort (69.4%) had Belgian nationality. Of the 396 from abroad, 345 patients (87%) were from the Netherlands. Table 1 illustrates the detailed patient demographics. All patients underwent a preoperative upper gastro-intestinal (GI) endoscopy, as well as upper GI contrast studies. Every case was assessed and approved by a multidisciplinary team prior to each operation.

**Table 1:** Demographic and operative data

	One-stage, band in situ	One-stage, without band	Two-stage	*p* value
	*N* = 840	*N* = 327	*N* = 128	
Age	44.5 ± 11.5	45.4 ± 10.0	42.1 ± 11.1	0.0212
Gender (M/F)	18%/82%	16%/84%	17%/83%	0.6837
Initial weight at LAGB	118.6 ± 22.7	113.6 ± 21.3	117.1 ± 21.1	0.0027
Initial BMI at LAGB	42.1 ± 6.9	40.9 ± 6.3	41.4 ± 6.0	0.0233
Weight at RYGB	110.8 ± 22.3	111.3 ± 21.7	110.2 ± 20.6	0.8872
BMI at RYGB	39.2 ± 6.7	40.04 ± 6.2	38.9 ± 6.1	0.1078

*M* male, *F* female, *LAGB* laparoscopic gastric banding, *LRYGB* laparoscopic Roux-en-Y gastric bypass, *BMI* body mass index

**Fig. 3:**
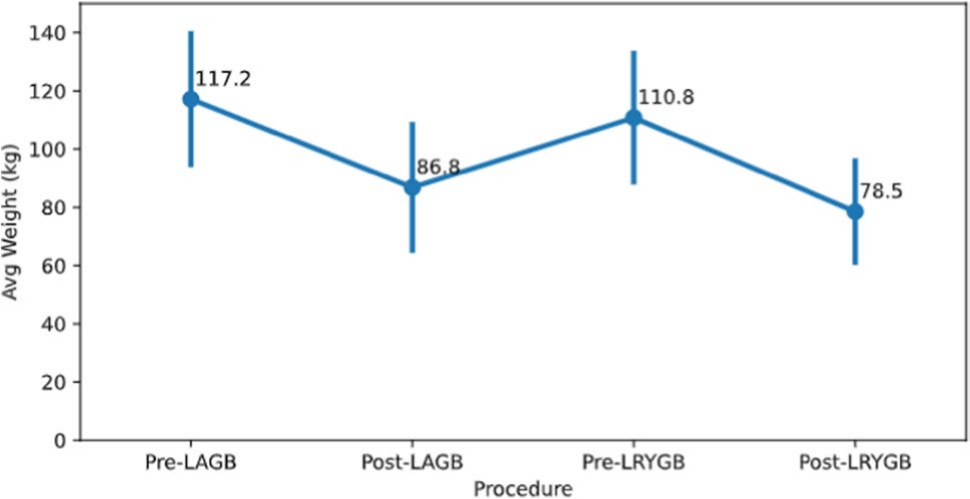
Weight evolution: pre-LAGB, post-LAGB, pre-LRYGB, and post-LRYGB at 1 year. *LAGB* laparoscopic gastric banding, *LRYGB* laparoscopic Roux-en-Y gastric bypass

The mean preoperative weight before LAGB placement was 117.2 ± 22.3 kg, corresponding with a mean initial BMI of 41.7 ± 6.7 kg/m^2^. This reduced to a mean nadir weight of 86.9 ± 21.5 kg and mean lowest BMI of 30.9 ± 6.7 kg/m^2^ post-banding. The mean preoperative BMI before conversion to Roux-en-Y gastric bypass was 39.4 ± 6.5 kg/m^2^. A detailed overview of the weight evolution is depicted in Fig. 3.

### Reasons for Conversion

In 396 patients (30.6%), there were band-related complications. Band intolerance was described in 798 patients (61.6%). Insufficient weight loss, defined as less than 25% excess weight loss (%EWL) according to the criteria of Reinhold [14], occurred in 13.7% (*n* = 177) of patients who underwent LAGB. The number of patients with less than 25% EWL increased to 72% (*n* = 932) prior to LRYGB.

### Perioperative Data

**Fig. 4:**
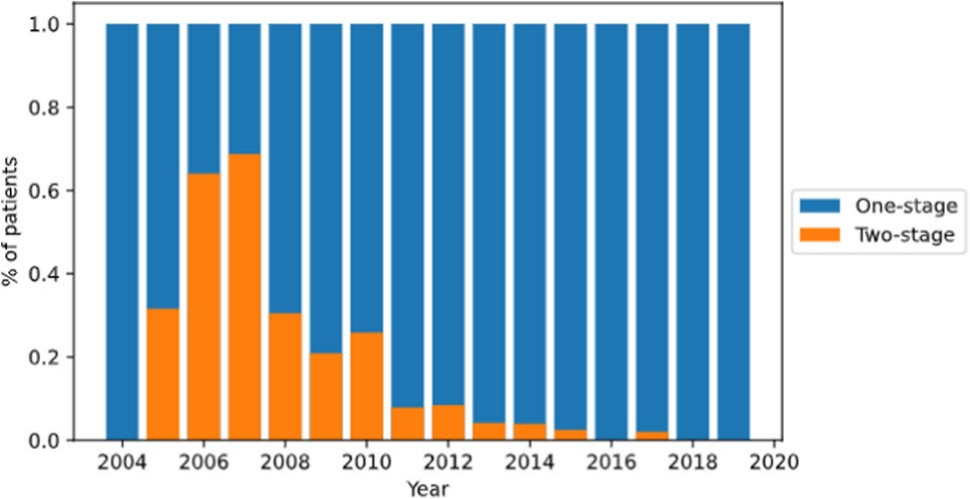
The effect of learning curve on one- and two-stage conversions

The placement of the band was performed in our center in 12.1% of patients, while 87.9% were initially operated elsewhere. In 99.1% of the cases, the band placement was performed laparoscopically, including via single incision operation technique. Twelve patients (0.9%) underwent an open banding procedure. At the time of referral for conversion to LRYGB, a previous reintervention (i.e., repositioning or re-banding) had occurred in 14.9% of patients. Figure 2 shows that a one-stage conversion was possible in 90.1% of patients (*n* = 1167). Of these procedures, the band had been left in place in 64.8% (*n* = 840) and others removed before referral to our hospital in 25.3% (*n* = 327). In 9.9% of cases (*n* = 128), a two-stage procedure occurred. In due course of our experience, there was an important increase of conversion ratio to one-stage probably due to increase of the learning curve (Fig. 4). In 24 patients (1.9%), we re-banded the gastric bypass.

### Complications and Mortality

Of the 1295 patients in our cohort, there was no mortality. There were 93 patients (7.2%) with an early (<30 days) complication. The most common complication was hemorrhage, either endoluminal (*n* = 26, 2.0%) or extraluminal (*n* = 13, 1.0%). Of the endoluminal bleedings, 14 patients were treated conservatively by means of careful hemodynamic monitoring with or without blood transfusion, while 10 patients underwent endoscopic treatment, and exceptionally, 2 patients required laparoscopic intervention; one required over suturing of a persistent bleeding spot at the gastrojejunostomy and the other needed evacuation of a blood bezoar at the entero-enterostomy. Of the extraluminal bleedings, the majority was managed conservatively (*n* = 10). Three patients required a laparoscopic revision with drainage of the blood cloths and control of the perioperative distinct bleeding location.

The reasons for intervention were lateral entrapment at a trocar site (*n* = 5), obstruction requiring adhesiolysis (*n* = 3), iatrogenic small bowel perforation (*n* = 1), internal hernia (*n* = 1), cholecystitis (*n* = 1), drainage of intra-abdominal seroma (*n* = 1), revision for removal of a rest piece of the band (*n* = 1), and one negative exploration for abdominal pain (*n* = 1). In total, a reoperation was necessary in 19 patients (1.4%). A postoperative infection was present in twenty-five patients (1.9%): 5 patients with pneumonia, 14 cases of wound infection, 2 cases of infected intra-abdominal hematoma, and lastly, there were 4 cases with fever and leucocytosis although no distinct cause could be identified.

**Fig. 5:**
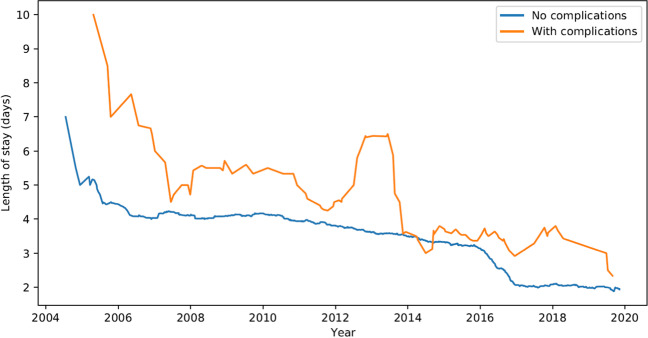
Length of hospital stay from 2004 to 2019

We had no leaks, neither at one of the anastomoses (gastrojejunostomy or entero-enterostomy), nor at the staple line dissection, although we had one case of iatrogenic small bowel lesion as described earlier. Table 2 summarizes the complications more in detail. There was no statistically significant difference in complication rates between the one- and two-stage groups. The mean postoperative hospital stay was 3.4 ± 1.1 days. Over the study period, an evolution to a shorter hospital stay is noticed (Fig. 5).

**Table 2:** Thirty-day complication rate

	One-stage (*n* = 1167)				Two-stage (*n* = 128)		*p* value	Overall complication rate (*n* = 1295)	
	Band in situ (*n* = 840)		Without band (*n* = 327)						
Thromboembolic event	3	0.4%	0	0.0%	0	0.0%	0.8394	3	0.2%
Infection	13	1.5%	7	2.1%	5	3.9%		25	1.9%
Abdominal	1		0		1		0.2772	2	
Wound	8		5		1		0.6533	14	
Unknown	2		1		1		0.5873	4	
Pneumonia	2		1		2		0.0766	5	
Acute cholecystitis	1	0.1%	0	0.0%	0	0.0%	0.7681	1	0.1%
Bleeding	28	3.3%	6	1.8%	5	3.9%		39	3.0%
Endoluminal	19		4		3		0.5035	26	
Extraluminal	9		2		2		0.6229	13	
Bowel perforation	1	0.1%	0	0.0%	0	0.0%	0.7681	1	0.1%
Lateral entrapment	2	0.2%	2	0.6%	1	0.8%	0.4891	5	0.4%
Internal herniation	1	0.1%	0	0.0%	0	0.0%	0.7681	1	0.1%
Obstruction	1	0.1%	1	0.3%	1	0.8%	0.3310	3	0.2%
Stenosis gastroenterostomy	1	0.1%	0	0.0%	0	0.0%	0.7681	1	0.1%
Extraction of corpus alienum	1	0.1%	0	0.0%	0	0.0%	0.7681	1	0.1%
Others	8	1.0%	4	1.2%	1	0.8%	0.8849	13	1.0%
Total	60	4.6%	20	1.5%	13	1.0%	0.3234	93	7.2%

### Weight Loss Outcomes

Overall, the mean nadir weight at 1-year follow-up is 79.2 kg, corresponding to a mean BMI of 28.0 kg/m^2^, a mean %EWL of 72.8%, and a mean %EBMIL of 87.0% as depicted in Table 3. There was no statistically significant difference in mean postoperative %EWL, %EBMIL, or %TWL between the groups. Seven hundred five (83.9%) patients in the one-stage group with band in situ, 291 (89%) patients in the one-stage group without band, and 103 (80.5%) patients in the two-stage group reached %EBMIL > 50.

**Table 3:** Weight loss outcomes

	One-stage, band in situ	One-stage, without band	Two-stage	*p* value	Total
Final weight	79.8 ± 17.8	77.4 ± 16.6	79.6 ± 18.4	0.1264	79.2 ± 17.6
Final BMI	28.2 ± 5.4	27.8 ± 4.7	28.1 ± 5.4	0.4383	28.0 ± 5.4
%EWL	72.5 ± 24.4	72.2 ± 24.8	72.5 ± 25.2	0.9776	72.8 ± 26.0
%EBMIL	86.9 ± 36.8	85.3 ± 36.7	86.7 ± 33.2	0.7830	87.0 ± 37.9
%TWL	32.2 ± 10.4	31.3 ± 10.7	31.5 ± 10.5	0.3570	32.1 ± 11.0

*BMI* body mass index, *EWL* excess weight loss, *EBMIL* excess body mass index loss, *TWL* total weight loss

## Discussion

Although acceptable weight loss (42.8% EWL) after 10 years of LAGB has been recently described by O’Brien et al., long-term failure and band-related complications in general have sealed the fate of the band as a successful primary bariatric surgical procedure [15]. The percentage of patients requiring band removal increases over time, from 5.6 to 59.4% [4, 5, 15, 16]. In conjunction, up to 60% of reoperation rates are reported [6, 7]. Hjorth et al. examined the incidence of revisional bariatric surgery over the 26-year follow-up of participants in the Swedish Obese Subjects (SOS) study and concluded that revisional surgery is most frequently seen following LAGB (40.7%) and the conversions were mainly to LRYGB [17].

As removal of the band will inevitably result in weight gain, the vast majority of patients opt for a conversion procedure. Currently, LRYGB and LSG are the most frequently performed revisional surgeries following band failure [10]. To date, however, there is no consensus regarding the preferred and most successful conversion method. Numerous results regarding LAGB revisional surgery have been published, but most studies have small sample sizes [18–32]. Our preference is a conversion to LRYGB, because it is believed to improve reflux and provide better weight loss due to the addition of moderate malabsorption to restriction. Many authors have favored conversion to LRYGB rather than LSG, claiming that weight loss is more efficient [9, 18, 33, 34], with a lower leakage rate [35]. To our knowledge, this study represents the largest series of RYGB after failed LAGB in a single center by the same surgeon so far, as a sequel of our previous article [11].

In our study, the incidence of early complications was 7.2% with a revision rate of 1.4%, which is generally lower than the vast majority of other authors have reported (Table 4). Only Spivak et al. reported a lower early complication rate of 3% in a series of 33 patients, with one patient undergoing open splenectomy for a bleeding spleen [36]. Noteworthy is the maintenance of zero percentage leakage rate and 30-day mortality in these series which is equivalent to our results in 2016, even though revisional bariatric surgery is technically more challenging. For example, in the systematic review of Pedziwiatr et al., the morbidity rate of the revisional gastric bypass was 18.6% which was significantly higher than in primary LRYGB [37]. Chansaenroj et al. compared 53 patients who underwent revisional LRYGB, laparoscopic single-anastomosis gastric bypass, and LSG following gastric banding and found 11.1% of early major complications in the group of LRYGB [38]. According to Coblijn et al., 8.5% of patients who underwent RYGB after LAGB experienced short-term problems, with wound infection being the most common [35]. In our opinion, performing a high volume of bariatric procedures with full standardization of every technical pre- and postoperative step is the key to success.

**Table 4:** Selected single- or multicenter studies reporting on 30-day morbidity, mortality, and reinterventions of LRYGB after failed LAGB

	Center	No of pts	OS/TS	Morbidity	Reinterventions	Mortality
Mognol et al. [25]	Single	70	47/23	14.3%	5.7%	0.0%
Hii et al. [26]	Single	82	64/18	46.3%	12.2%	0.0%
Aarts et al. [27]	Single	195	195/0	8.7%	4.1%	N/A
Al-Kurd et al. [28]	Single	161	121/40	6.6% (OS), 10% (TS)	4.1% (OS), 7.5% (TS)	N/A
Weber et al. [29]	Single	32	32/0	12.5%	6.3%	0.0%
Moore et al. [30]	Single	26	24/2	11.0%	3.8%	0.0%
Spivak et al. [36]	Single	33	33/0	3.0%	3.0%	0.0%
Apers et al. [31]	Single	86	50/36	34%	N/A	0.0%
Stroh et al. [32]	Multi	379	263/116	10.3% (OS), 12.1% (TS)	N/A	0.7%/0.0%

*N/A* not applicable, *OS/TS* one-stage/two-stage

Whether sleeve gastrectomy is a valuable alternative conversion procedure for failed gastric banding was not the scope of this study. However, several systematic reviews find comparable rates of complications, morbidity, and mortality between the revisional LRYGB and LSG [39–41]. On the other hand, both Paniolos et al. and Janik et al. found that patients undergoing LRYGB following gastric banding removal experience higher complication rates and more need for additional early procedures compared with LSG [42, 43]. However, both systematic reviews report only on a single stage conversion without considering that a two-stage procedure would be safer in certain conditions as suspected leak or migrated band to avoid early complications.

Noteworthy is an evolution in our study group regarding the length of hospital stay and performing the secondary LRYGB in one stage, as the surgical exposure and experience in a high-volume center increase. A mean length of hospital stay of 4 days is illustrated in 2004, which decreases to a mean of 2 days from 2016 onwards (Fig. 5). Similarly, only one conversion (1.4%) in two stages was performed since 2016, while the highest peak of two-stage procedures was 58.9% in 2007 (Fig. 4).

Our findings confirm no significant difference in complication rate between one-stage and two-stage procedures, as was already described by Debergh et al. in the precursor of this study [11]. Similarly, Alratrout et al. reported 14.02% (15/107 patients) overall complications in the one-step group versus 7.89% (6/76 patients) in the two-step group with no statistical significance [41]. A recent systematic review of the literature by Marion et al. with a total of 3895 patients concludes that conversion of failed LAGB to LRYGB or LSG can be safely performed in both one- and two-step approaches [44]. The importance of patient selection is underlined by Pujol-Rafols et al., where a one-stage procedure is suggested in the absence of major mechanical band failure and a two-stage procedure in the case of band slippage or erosion [45]. Overall, 84% (1099 patients) reached a %EBMIL >50. No difference in weight loss was observed between our groups compared to what has been published earlier in revisional surgery of gastric banding; a %EWL at 12 months of 72.8% is highly satisfactory (Table 5). Studies with small sample sizes reported %EWL at 12 months between 45.6 and 65.8% for LRYGB after failed LAGB [18–24].

**Table 5:** Selected papers reporting on 1-year %EWL of LRYGB and LSG after failed gastric banding

	*N* (LRYGB/LSG)	%EWL	
		LRYGB	LSG
Angrisani et al. [19]	24/27	65.8	67.2
Liu et al. [20]	40/48	56.4	49.8
Marin-Perez et al. [18]	39/20	59	35
Yeung et al. [21]	27/47	50	45.5
Khoursheed et al. [22]	53/42	45.6	47.5
Carr et al. [23]	64/25	52.1	44.1
Gonzalez-Heredia et al. [24]	12/26	46	64.4

The current study has some limitations. Although data were collected prospectively, they were analyzed retrospectively. As such, some data were lacking and data interpretation might be prone to selection bias, which is inherent to retrospective analysis. Second, because of the overall limited number of complications, the study might have lacked the power to discover differences between the groups. Finally, there was no comparison with other plausible procedures, such as sleeve gastrectomy and single anastomosis gastric bypass. This survey analyzed the short-term morbidity and mortality of the LRYGB solely as a revisional procedure, but whether Roux-en-Y gastric bypass is the best revisional procedure after failed gastric banding needs to be examined by the long-term outcome data on morbidity, mortality, weight loss, and comorbidities as well. These long-term results from the same prospectively collected database are to be expected soon. However, the main strength of this study is the size of the study population of this monocentric study, implementing power to our results.

## Conclusion

With experienced hands and full standardization of the revisional procedure, conversion of failed LAGB to LRYGB can be considered a safe and effective option with a low complication rate and good weight loss outcomes at 1 year. This single-center series of 1295 patients who underwent LRYGB after gastric banding reported only 7.2% of early complications with a surgical revision rate of 1.4%, no leakage, and zero mortality. Furthermore, our findings confirm that conversion in one stage has no significant difference in early outcomes compared to the two-step approach. Reviewing our data, we believe one-stage conversion will be performed more often as the experience of the surgeon increases. Future work will reveal the long-term outcomes of our study group.
